# Defective monocyte oxidative burst predicts infection in alcoholic hepatitis and is associated with reduced expression of NADPH oxidase

**DOI:** 10.1136/gutjnl-2015-310378

**Published:** 2016-02-09

**Authors:** Nikhil Vergis, Wafa Khamri, Kylie Beale, Fouzia Sadiq, Mina O Aletrari, Celia Moore, Stephen R Atkinson, Christine Bernsmeier, Lucia A Possamai, Gemma Petts, Jennifer M Ryan, Robin D Abeles, Sarah James, Matthew Foxton, Brian Hogan, Graham R Foster, Alastair J O'Brien, Yun Ma, Debbie L Shawcross, Julia A Wendon, Charalambos G Antoniades, Mark R Thursz

**Affiliations:** 1 Department of Hepatology and Gastroenterology, Imperial College, London, UK; 2 Department of Hepatology, King's College Hospital, Institute of Liver Studies, London, UK; 3 Chelsea and Westminster Hospital, London, UK; 4 Department of Hepatology, Royal Free Hospital, London, UK; 5 Department of Gastroenterology, Royal London Hospital, London, UK; 6 Department of Hepatology, University College, London, UK

**Keywords:** BACTERIAL INFECTION, ALCOHOLIC LIVER DISEASE, IMMUNOLOGY IN HEPATOLOGY

## Abstract

**Objective:**

In order to explain the increased susceptibility to serious infection in alcoholic hepatitis, we evaluated monocyte phagocytosis, aberrations of associated signalling pathways and their reversibility, and whether phagocytic defects could predict subsequent infection.

**Design:**

Monocytes were identified from blood samples of 42 patients with severe alcoholic hepatitis using monoclonal antibody to CD14. Phagocytosis and monocyte oxidative burst (MOB) were measured ex vivo using flow cytometry, luminometry and bacterial killing assays. Defects were related to the subsequent development of infection. Intracellular signalling pathways were investigated using western blotting and PCR. Interferon-γ (IFN-γ) was evaluated for its therapeutic potential in reversing phagocytic defects. Paired longitudinal samples were used to evaluate the effect of in vivo prednisolone therapy.

**Results:**

MOB, production of superoxide and bacterial killing in response to *Escherichia coli* were markedly impaired in patients with alcoholic hepatitis. Pretreatment MOB predicted development of infection within two weeks with sensitivity and specificity that were superior to available clinical markers. Accordingly, defective MOB was associated with death at 28 and 90 days. Expression of the gp91*^phox^* subunit of nicotinamide adenine dinucleotide phosphate (NADPH) oxidase was reduced in patients with alcoholic hepatitis demonstrating defective MOB. Monocytes were refractory to IFN-γ stimulation and showed high levels of a negative regulator of cytokine signalling, suppressor of cytokine signalling-1. MOB was unaffected by 7 days in vivo prednisolone therapy.

**Conclusions:**

Monocyte oxidative burst and bacterial killing is impaired in alcoholic hepatitis while bacterial uptake by phagocytosis is preserved. Defective MOB is associated with reduced expression of NADPH oxidase in these patients and predicts the development of infection and death.

**Significance of this study**

**What is already known on this subject?**

- Infection is common during severe alcoholic hepatitis (SAH) and can cause death.
- Routine prescribing of antibiotics to all patients with SAH is not advocated, however, and it is, therefore, important to find biomarkers that can predict the development of infection.
- Bacterial killing is effected by innate immune cells through phagocytosis, but the precise phagocytic defects that underlie increased susceptibility to infection remain unclear, with no studies of monocyte phagocytosis in SAH to date.
- Interferon-γ (IFN-γ) has been proposed as a candidate to reverse neutrophil phagocytic dysfunction.

**What are the new findings?**

- Monocytes from patients with SAH are able to take up *Escherichia coli* by phagocytosis, but defective oxidative burst results in impaired bacterial killing.
- Defective monocyte oxidative burst (MOB) predicted the development of infection within the subsequent 2 weeks and death by 28 days.
- Impaired MOB was associated with reduced expression of nicotinamide adenine dinucleotide phosphate oxidase, an enzyme that generates the superoxide radicals required for bacterial killing.
- Defective MOB could not be reversed by IFN-γ, and this resistance was associated with increased expression of the negative regulator of Janus Kinase-signal transducer and activator of transcription signalling, suppressor of cytokine signalling-1.

**How might it impact on clinical practice in the foreseeable future?**

- MOB may be used as a biomarker to predict the development of infection in patients with SAH and rationalise prescribing of prophylactic antibiotics.
- Targeting defective MOB with molecular or biochemical techniques may reduce susceptibility to infection and death in patients with SAH.

## Introduction

Alcoholic hepatitis is the most florid form of alcoholic liver disease. Severe alcoholic hepatitis (SAH) develops after heavy and prolonged alcohol consumption and is associated with high short-term mortality when Maddrey's discriminant function (MDF) is >32.^1^

Infection is an important contributor to mortality in SAH. Twenty-five per cent of patients are admitted with infection, and a further 25% develop nosocomial infections during their hospital stay.^2^ Nosocomial infection more than doubles 60-day mortality in patients treated with corticosteroids.^2^ This susceptibility to infection represents an immune paresis that, as yet, is only partially explained. Moreover, there is no biomarker currently available that is able to predict the development of infection in order to guide antibiotic prescribing and prevent death from infection in SAH.

Monocytes and neutrophils engulf invading bacteria via phagocytosis. Phagocytosed bacteria are subsequently killed by the generation of superoxide radicals (O_2_^−^) in a process known as oxidative burst. Impairments in circulating neutrophil phagocytosis and oxidative burst have been reported in SAH and are associated with increased mortality.^3–5^ Neutrophils from patients with SAH have elevated baseline reactive oxygen species (ROS) levels^4^ and reduced phagocytic capabilities.^3^ ^5^ ^6^ One study suggested that reduced production of the immunostimulatory cytokine interferon-γ (IFN-γ) from T cells is the cause of impaired neutrophil oxidative burst.^6^

Interestingly, defective phagocyte oxidative burst is the hallmark of the inherited condition chronic granulomatous disease (CGD), for which prophylactic IFN-γ therapy can reduce the number of serious infections and hospitalisations.^7^ However, no study to date has examined the utility of IFN-γ in improving phagocyte oxidative burst in SAH. Similarly, no study has examined the impact of prednisolone therapy on oxidative burst, despite the common use of this drug as treatment for SAH and its frequently cited association with nosocomial infection. Furthermore, although monocyte dysfunction is reported to contribute to immune paresis in allied liver failure syndromes such as acute on chronic liver failure and acute liver failure,^8–10^ there are no data describing monocyte phagocytic capabilities in SAH.

The present study aims to evaluate phagocytic function of monocytes during SAH and relate defects to the development of infection. In addition, the impact of in vivo prednisolone and the reversibility of ex vivo phagocytic dysfunction with in vitro IFN-γ were tested.

## Materials and methods

### Patients and sampling

In total, 101 subjects were recruited to the study from six hospitals in London, UK, between January 2011 and September 2014.

Patients were categorised as follows: 42 patients with SAH; 25 compensated alcohol-related cirrhotic patients, divided into patients who had been abstinent for at least 6 months (chronic liver disease [CLD], n=11) and patients who had been actively drinking within the preceding 6 months (drinking chronic liver disease [dCLD], n=14); and 34 healthy controls. All patients with SAH had an alcohol consumption of >80 g/day (men) or >60 g/day (women) immediately prior to hospital admission; had bilirubin >80 µmol/L; and MDF ≥32. In addition, patients with SAH satisfied clinical diagnostic criteria described in the *Steroids or Pentoxifylline for Alcoholic Hepatitis* (STOPAH) clinical trial protocol.^11^ In particular, the attending physician controlled any infection with intravenous antibiotics for at least 48 h before entering the patient into the study. These criteria are listed in online supplementary table S1.

10.1136/gutjnl-2015-310378.supp1 Supplementary materials

10.1136/gutjnl-2015-310378.supp2 Supplementary materials

Patients with chronic liver disease were recruited from outpatient clinics and had cirrhosis diagnosed by previous liver biopsy or clinical presentation with typical ultrasound or CT imaging. HC were members of clinical or university staff at St Mary's Hospital with no evidence of liver dysfunction.

### Definition of infection

Patients were deemed to have developed infection if any of the following criteria were met in line with recently published criteria: (i) positive blood cultures; (ii) ascitic neutrophil count >250 cm^−3^; (iii) consolidation on chest radiograph in conjunction with respiratory signs or laboratory markers of infection; (iv) diarrhoea with positive stool cultures for pathogenic bacteria; (v) cellulitis with fever or laboratory signs of infection; (vi) positive urine culture; (v) intra-abdominal infections: diverticulitis, appendicitis and cholangitis; and (vi) secondary bacterial peritonitis: ascitic neutrophils >250 cm^−3^ in the presence of intra-abdominal source of peritonitis and multiple organisms cultured from ascitic fluid.^12^

### Monocyte phagocytosis and oxidative burst assays

Monocyte oxidative burst (MOB) was assessed ex vivo using the Phagoburst kit according to the manufacturer’s instructions (Glycotrope, Germany)*.* In brief, 100uL whole blood was incubated with (test condition) and without (control condition) 2x10^7^ E. coli for 20 min at 37°C. 20 μL 1,2,3-dihydrorhodamine (1,2,3DHR) was then added to each condition for a further 20 min at 37°C, and the oxidation to rhodamine within CD14^+^ monocytes was measured by flow cytometry. Test responses were compared with control responses in order to deduce the MOB response to the bacteria. Phagocytosis was measured using the pHRodo kit (ThermoFisher Scientific, UK) according to the manufacturer's instructions.

### Monocyte isolation

Peripheral blood mononuclear cells (PBMCs) were isolated from heparinised fresh whole blood after Ficoll density gradient centrifugation according to established protocol.^8^ Monocytes were then isolated using the Pan Monocyte Isolation Kit (Miltenyi Biotec, Germany) according to the manufacturer's instructions. A representative figure illustrating monocyte purity is given in online supplementary figure S1.

### Monocyte superoxide production

Purified monocytes were co-cultured for 40 min with *E. coli* at a monocyte: *E. coli* ratio of 1:100. The cell suspension was then mixed with Diogenes reagent (National Diagnostics, USA) according to the manufacturer's instructions and superoxide was quantified by recording luminescence (relative light units) using a luminometer (FLUOstar OPTIMA, BMG Labtech) according to the manufacturer's instructions.

### Bacterial killing assay

Bacterial killing in supernatant and lysate fractions was measured as previously described.^13^ Briefly, isolated monocytes were incubated with *E. coli* (K12, Stratagene) at a 1:100 ratio in Roswell Park Memorial Institute medium (RPMI) (Sigma, UK) supplemented with 10% healthy AB serum (Sigma). After 40 min, cooling to 0°C stopped the reaction and supernatants were aspirated. The monocyte cell pellet was lysed in distilled water pH 11 according to published literature.^14^ Supernatant and lysate fractions were plated separately onto agar plates (Sigma) at dilutions of 1:10, 1:100 and 1:1000 and colony-forming units were counted after 18 h incubation at 37°C as previously described.^13^

### IFN-γ and prednisolone co-culture

PBMCs were incubated for 24 h in RPMI (Sigma) supplemented with 10% autologous patient serum and 50 ng/mL IFN-γ or 10 μg/mL prednisolone that had been dissolved at 37° for 24 h prior. The ability of CD14+ monocytes to oxidise 1,2,3-DHR to rhodamine in response to incubation with *E. coli* was measured using modifications to the flow cytometry-based Phagoburst assay (Glycotrope).

### Western blotting of monocyte G6PDH, pSTAT-1, SOCS-1, gp91*^***phox***^* and p47^***phox***^ proteins

Isolated monocytes were rested for 4 h in X-VIVO medium (Lonza, Switzerland) and then stimulated with 50 ng/mL IFN-γ for 20 min. Cells were then lysed with ice-cold nonyl phenoxypolyethoxylethanol-40 buffer (Invitrogen, UK) containing proteinase inhibitors (Sigma). Protein was separated on 4–12% Bio-Rad gels (Bio-Rad, UK) and transferred to polyvinylidene fluoride membranes. Western blotting was performed using monoclonal antibodies against glucose-6 phosphate dehydrogenase (G6PDH) (Abcam, UK), gp91*^phox^* (Abcam), phospho-signal transducer and activator of transcription-1 (pSTAT-1) (BD Bioscience, UK), p47*^phox^* and Suppressor of Cytokine Signalling-1 (SOCS-1) (Santa Cruz, USA).

### Paired longitudinal samples

Ex vivo MOB was measured before (day 0 MOB) and 7 days (day 7 MOB) after the start of therapy as determined by the double-blind randomised design of the STOPAH trial. After completion of the STOPAH trial, treatment allocation data were released and used to compare results from patients given oral prednisolone to patients not given oral prednisolone.

### Statistical analysis

Continuous data are expressed as median, IQR or mean (±SD) as appropriate. The significance of differences between medians or means was tested using Mann–Whitney test or Wilcoxon-pairs signed rank test for non-parametric data and paired or unpaired Student's t tests for parametric data. Spearman's rank tests explored correlation. ORs are expressed with 95% CIs in parentheses. p Values are two-tailed except where specified, and p<0.05 was adopted as the threshold for statistical significance. Data were analysed and graphs were drawn using Prism V.6.0 (GraphPad, USA).

## Results

### Patient characteristics

Baseline patient and clinical characteristics are summarised in tables 1 and 2, respectively.

**Table 1:** Baseline patient characteristics

	SAH	CLD	HC
Age (years)	47 (41–56)	49 (47–56)	42 (36–53)
Male (%)	65	70	30
MELD	24 (22–27)	12 (9–15)	n/a
CTP score (class)	10 (C)	7 (B)	n/a
INR	1.8 (1.5–2.0)	1.2 (1.2–1.4)	n/a
Bilirubin (μmol/L)	318 (200–460)	26 (8–44)	n/a
Albumin (g/L)	24 (19–33)	31 (28–36)	n/a
White cell count (×10^9^/L)	8.9 (5.6–13.3)	4.9 (3.7–6.1)	n/a
Monocyte count (×10^9^/L)	1.0 (0.6–1.5)	0.5 (0.4–0.7)	n/a
Neutrophil count (×10^9^/L)	6.9 (3.8–10.3)	2.5 (2.4–3.7)	n/a
Alanine transferase (IU/L)	42 (30–79)	21 (17–36)	n/a
Aspartate transaminase (IU/L)	127 (100–164)	59 (34–79)	n/a
Serum creatinine (μmol/L)	69 (63–104)	63 (57–71)	n/a

Median average values (IQR) are shown unless otherwise stated.

CTP, Child-Turcotte-Pugh score; HC, healthy control; INR, international normalised ratio; MELD, Model for End-Stage Liver Disease; SAH, severe alcoholic hepatitis.

**Table 2:** Clinical characteristics of severe alcoholic hepatitis study participants

Maddrey's discriminant function (IQR)	57 (41–76)
Prednisolone therapy	52% (22/42)
No prednisolone therapy	48% (20/42)
Lille score^15^ (IQR)	0.4 (0.12–0.65)
Patients receiving antibiotics before initial sampling	50%
Patients with infection* before initial sampling	21%
Patients receiving new or a change of antibiotics within 2 weeks of initial sampling	50%
Patients developing nosocomial infection within 2 weeks of initial sampling (infected patients*)	40%
28-Day mortality of infected patients*	35%
28-Day mortality of patients who were not infected*	0%
90-Day mortality of infected patients*	63%
90-Day mortality of patients who were not infected*	15%

Values are median average (IQR) unless otherwise stated.

*Infection is defined according to consensus criteria.^12^

Patients were sampled at a median 5 (3–8) days after admission to hospital. Therapy was determined by randomisation within the STOPAH clinical trial in 74% (31/42) of patients and by clinical judgement in patients not participating in the clinical trial. In 22 cases, a further follow-up sample was obtained 7 days after the institution of therapy. The types of infection acquired are given in online supplementary table S2; pneumonia was the most common and accounted for 59%, followed by infection of the urinary tract at 18% of all infections.

### Phagocytosis of whole bacteria is preserved in SAH

**Figure 1:**
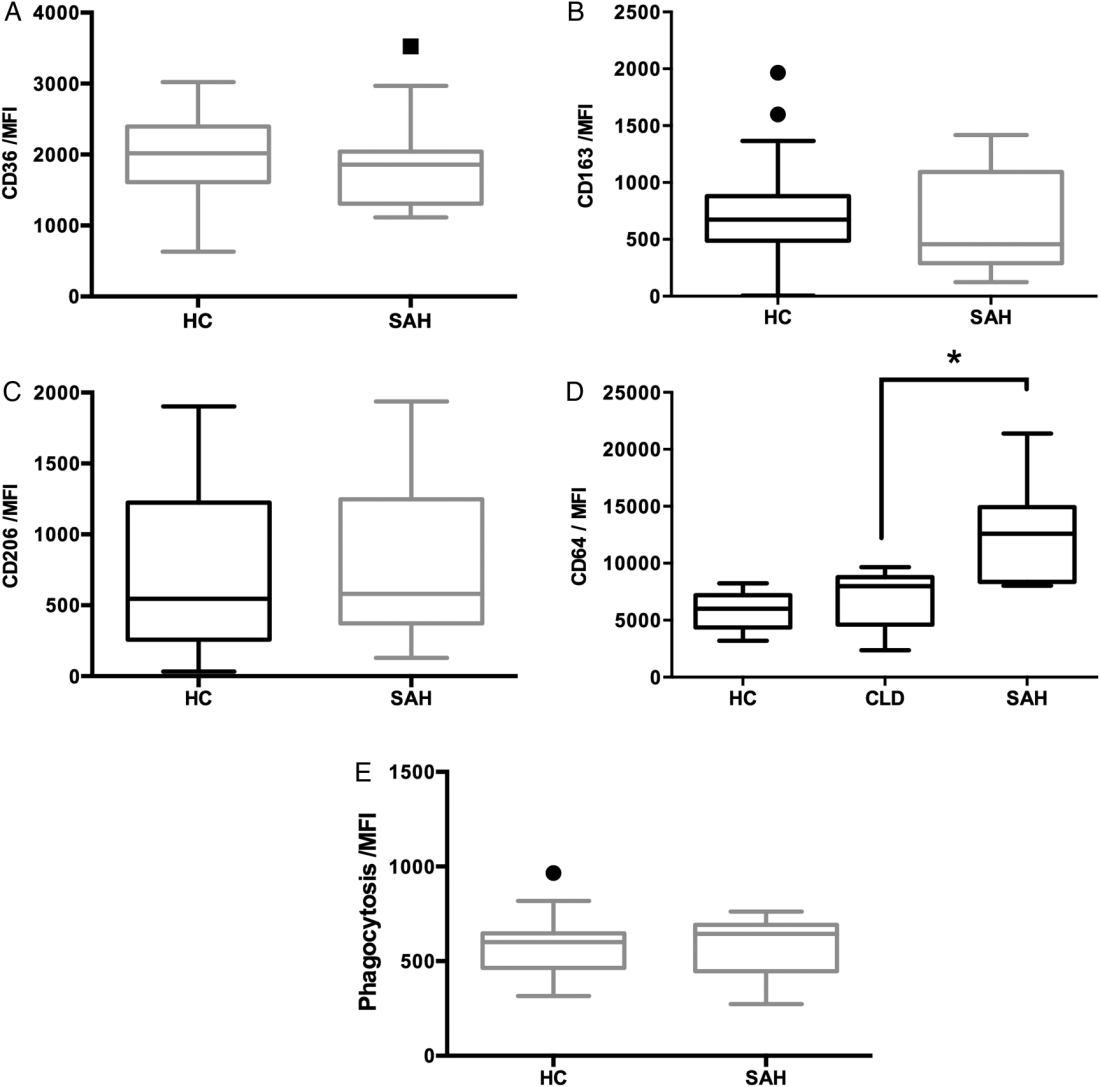
Uptake of bacteria by phagocytosis was similar between severe alcoholic hepatitis (SAH) and healthy monocytes. (A–C) Scavenger receptor expression was equivalent on SAH monocytes. (D) FcγR expression was increased in SAH compared with healthy control (HC) and CLD; (E) phagocytosis is preserved in SAH monocytes.

In order to examine phagocytic function of circulating monocytes in SAH, the expression of monocyte surface Fcγ-receptor CD64 and scavenger receptors (CD36, CD163 and CD206) were determined. While the expression of scavenger receptors in SAH was equivalent to HC (figure 1A–C), expression of Fcγ-receptor CD64 was increased in SAH compared with chronic liver disease (CLD) and HC (figure 1D). Accordingly, assays using the pHrodo technique showed that monocytes from patients with SAH were able to phagocytose *E. coli* as well as HC (figure 1E).

### MOB and bacterial killing are impaired in SAH

**Figure 2:**
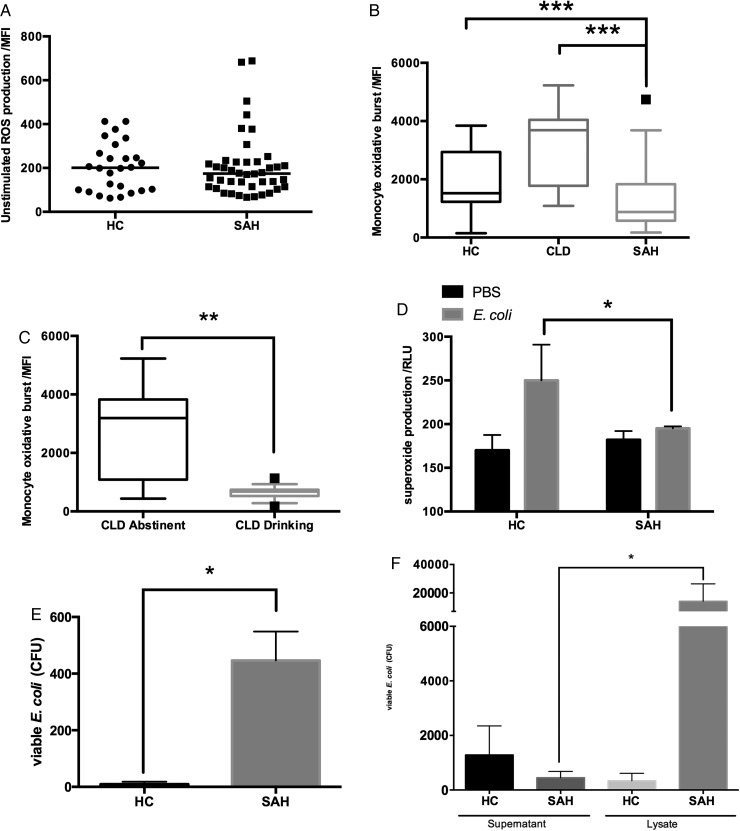
Monocyte oxidative burst (MOB) and bacterial killing is impaired in patients with severe alcoholic hepatitis (SAH). (A) The production of reactive oxygen species (ROS) at rest was similar between SAH and healthy control (HC) monocytes; (B) MOB in response to *Escherichia coli* is impaired in SAH compared with HC and CLD; (C) impaired MOB is also seen in cirrhotic patients who were actively drinking at the time of sampling compared with abstinent cirrhotic patients; (D) impaired MOB corresponded to a reduction in the production of superoxide radicals in response to *E. coli* from SAH monocytes; (E) killing of phagocytosed bacteria is reduced in SAH compared with HC monocytes; (F) far more *E. coli* were enumerated from the lysate (intracellular fraction) of SAH monocytes compared with the respective supernatant (extracellular fraction).

Intracellular killing of internalised bacteria using ROS was quantified by measuring 1,2,3DHR oxidation to fluorogenic rhodamine. The unstimulated level of ROS production in circulating monocytes from patients with SAH was similar to that found in HC (figure 2A). However, in response to *E. coli,* MOB was markedly impaired in patients with SAH compared with CLD and HC (figure 2B). Interestingly, defective MOB was also noted in actively drinking cirrhotic patients compared with abstinent cirrhotic patients (figure 2C).

The impaired ROS generation to *E. coli* in patients with SAH corresponded to a specific reduction in the production of superoxide (O_2_^−^) (figure 2D). Moreover, impaired MOB resulted in defective intracellular killing of bacteria. Increased numbers of viable *E. coli* were enumerated from lysates of monocytes from patients with SAH compared with HC monocytes (figure 2E). In particular, there were more colonies of *E. coli* enumerated from the lysates of patients with SAH compared with the corresponding supernatant, a phenomenon not seen in HC, supporting the hypothesis of adequate phagocytosis but defective intracellular bacterial killing in SAH monocytes (figure 2F).

### Defective MOB is associated with increased risk of developing infection

In view of previous data suggesting that the risk of contracting infection in SAH is dependent on liver function,^2^ we explored the relationship between liver function, systemic inflammation and MOB in patients with SAH.

**Figure 3:**
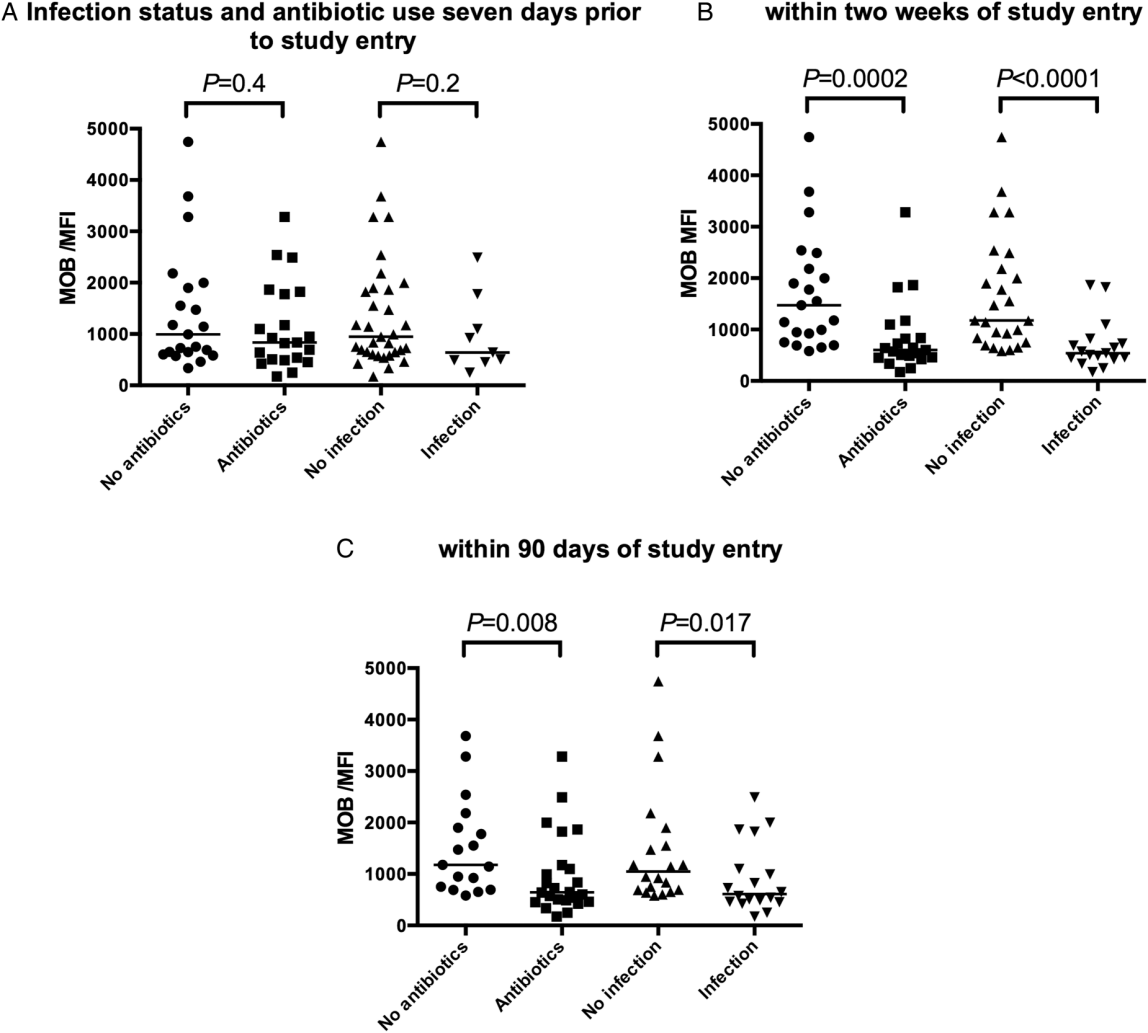
Impaired monocyte oxidative burst (MOB) predicts the subsequent development of infection within 2 weeks. (A) Prior prescription of intravenous antibiotics did not affect severe alcoholic hepatitis (SAH) MOB; (B) patients with SAH who developed infection within 2 weeks of sampling had a lower pretreatment MOB compared with patients who did not develop infection; (C) patients who developed infection within 90 days of sampling had a lower pretreatment MOB than patients who did not develop infection.

Prior treatment with systemic antibiotics had no impact on MOB in patients with SAH (figure 3A). Day 0 MOB correlated inversely with white cell count (WCC) (r=−0.5, p=0.001), C-reactive protein (CRP) (r=−0.4, p=0.01) and procalcitonin (PCT) (r=−0.37, p=0.02) but there was no correlation with either static or dynamic markers of liver function such as serum bilirubin, MDF, Model for End-Stage Liver Disease, early change in bilirubin level or Lille model.^15^ In contrast, a strong association between ex vivo MOB and the subsequent development of infection within 14 days was detected (figure 3B). This association remained statistically significant at 90 days (figure 3C).

SAH MOB had a broad IQR that overlapped that of HC (580–1832 median fluorescence intensity (MFI) vs 1230–2939 MFI). A range of MOB cut-points were tested and sensitivity and specificity values for predicting the development of infection within 2 weeks at each are given in table 3.

**Table 3:** Sensitivity and specificity of monocyte oxidative burst for predicting the subsequent development of infection using a range of cut-points

	Sensitivity (%)	Specificity (%)
<25th centile of HC	48	88
<50th centile of SAH	72	82
<25th centile of SAH	100	59

HC, healthy control; SAH, severe alcoholic hepatitis.

**Figure 4:**
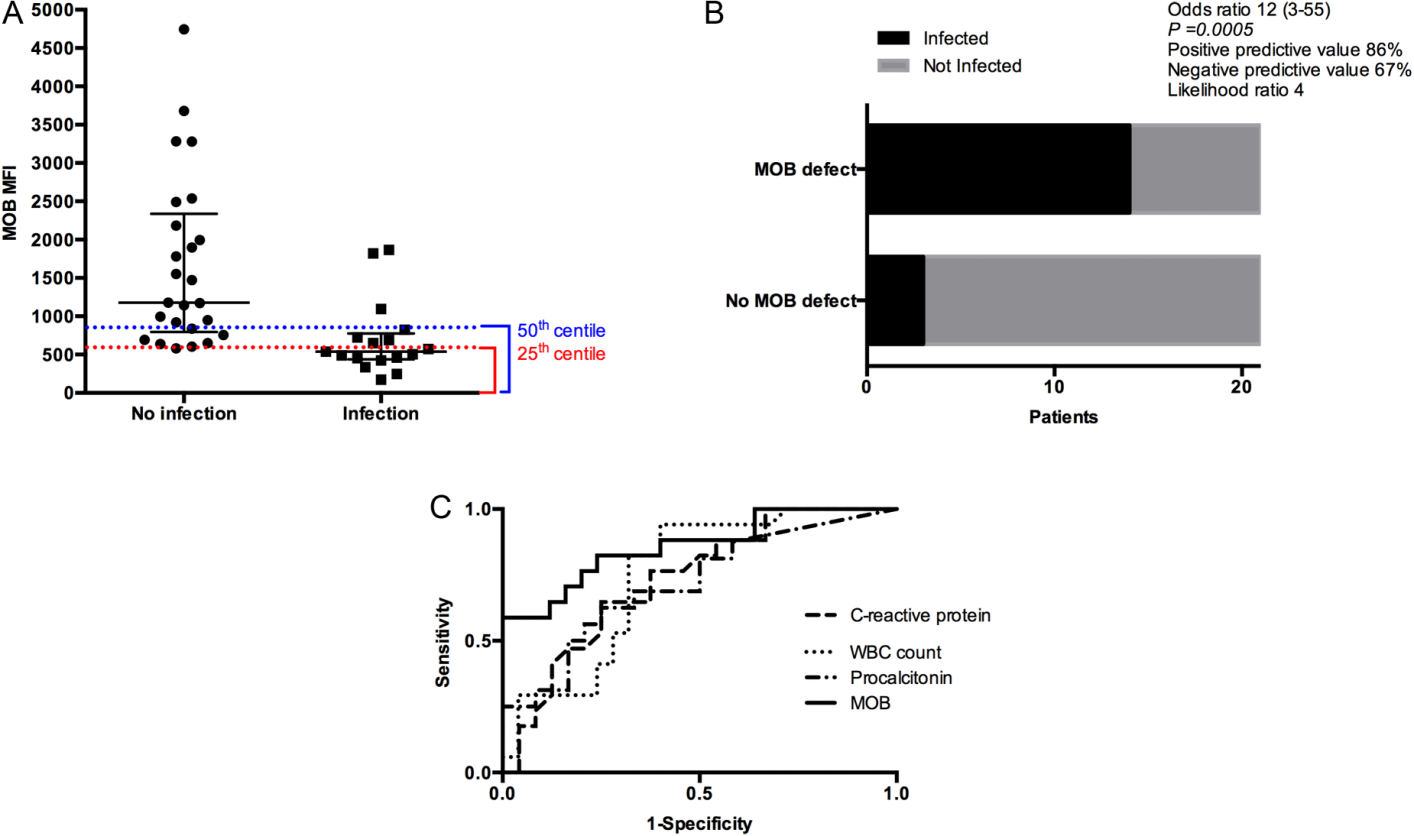
Monocyte oxidative burst (MOB) <50th centile (MOB defect) can identify patients likely to develop infection more accurately than conventional clinical markers of infection. (A) Cut-off values at the 50th and 25th centiles of severe alcoholic hepatitis (SAH) MOB and their association with the development of infection within the subsequent 2 weeks; (B) OR of MOB <50th centile (MOB defect) for predicting infection within 2 weeks; (C) receiver operating characteristic curve (ROC) curve for MOB and the development of infection within the subsequent 2 weeks; area under ROC (AUROC) is 0.86 (p<0.0001). For comparison, the ROC curves for C-reactive protein (CRP), white cell count (WCC) and procalcitonin in predicting the subsequent development of infection are included (AUROC 0.73, 0.75 and 0.72; p=0.019, 0.007 and 0.02, respectively).

A MOB cut-point at the 50th centile of SAH values had the greatest sensitivity and specificity for predicting the development of new infection (positive predictive value 0.86 (0.64–0.97); area under receiver operating characteristic curve (AUROC) 0.86 (0.74–0.98; p<0.0001)) (figure 4A–C). This was superior to AUROC values for WCC, CRP and PCT (0.75, 0.73 and 0.72, respectively).

The development of new infection within the first 2 weeks was associated with an increased risk of death at 28 and 90 days (OR 48 (3–918); p<0.0001 and OR 9 (2–46); p=0.003, respectively). Accordingly, day 0 MOB<50th centile, hereafter termed *MOB defect*, was associated with mortality at 28 and 90 days (OR 6.0 and 3.5, one-tailed p=0.044 and 0.041, respectively).

### MOB defect increases susceptibility to catalase-positive organisms

Bacteria that are able to use the enzyme catalase to defend against H_2_O_2_ mediated attack during phagocyte oxidative burst are known as catalase-positive organisms. We next sought to understand whether patients with MOB defect are more susceptible to catalase-positive organisms compared with patients without MOB defect.

**Figure 5:**
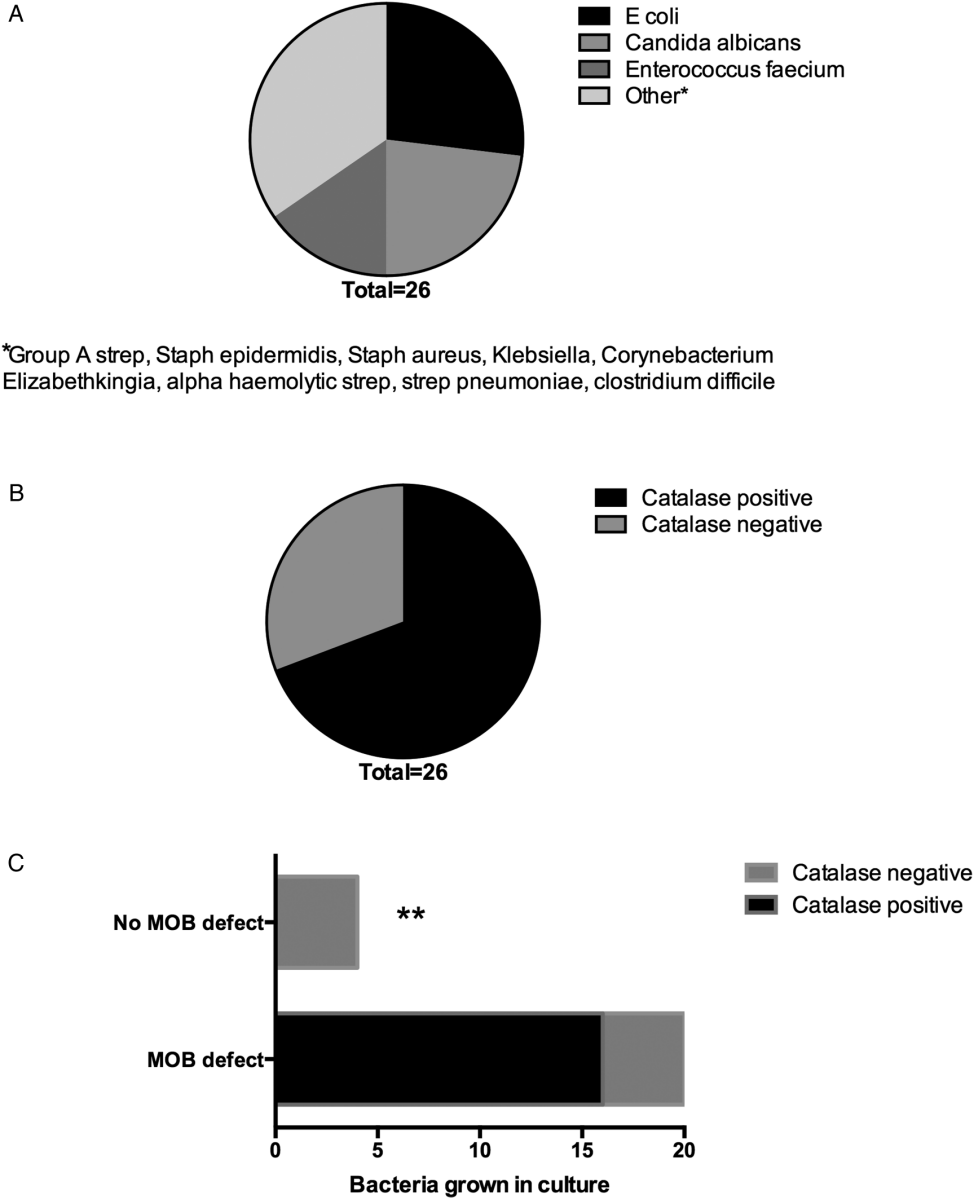
Patients with severe alcoholic hepatitis (SAH) with monocyte oxidative burst (MOB) defect are more susceptible to catalase-positive organisms: (A) organisms grown by culture from patients with SAH recruited to the study; (B) catalase status of organisms grown; (C) susceptibility of patients with SAH with MOB defect to catalase-positive organisms compared with patients with SAH without MOB defect.

*E. coli* was the organism grown most frequently by culture, followed by *Candida albicans*, and together these two organisms comprised 50% of all positive cultures (figure 5A). The majority of organisms grown by culture in patients with SAH were catalase positive (figure 5B). All of the catalase-positive organisms were grown in samples from patients with defective MOB; in contrast, just 4 of the 8 (50%) samples that grew catalase-negative organisms came from patients with defective MOB. Conversely, none of the patients with SAH with MOB >50th centile were infected by catalase-positive organisms (figure 5C). The OR of MOB defect predicting subsequent infection with a catalase-positive organism was 33 (p=0.007).

### In vivo prednisolone therapy does not depress MOB

We sought to determine whether the increased rate of infection seen in patients treated with prednisolone in the STOPAH trial^16^ could be attributed to an effect of prednisolone on MOB. We, therefore, measured MOB in sequential samples in patients with SAH treated with or without prednisolone.

**Figure 6:**
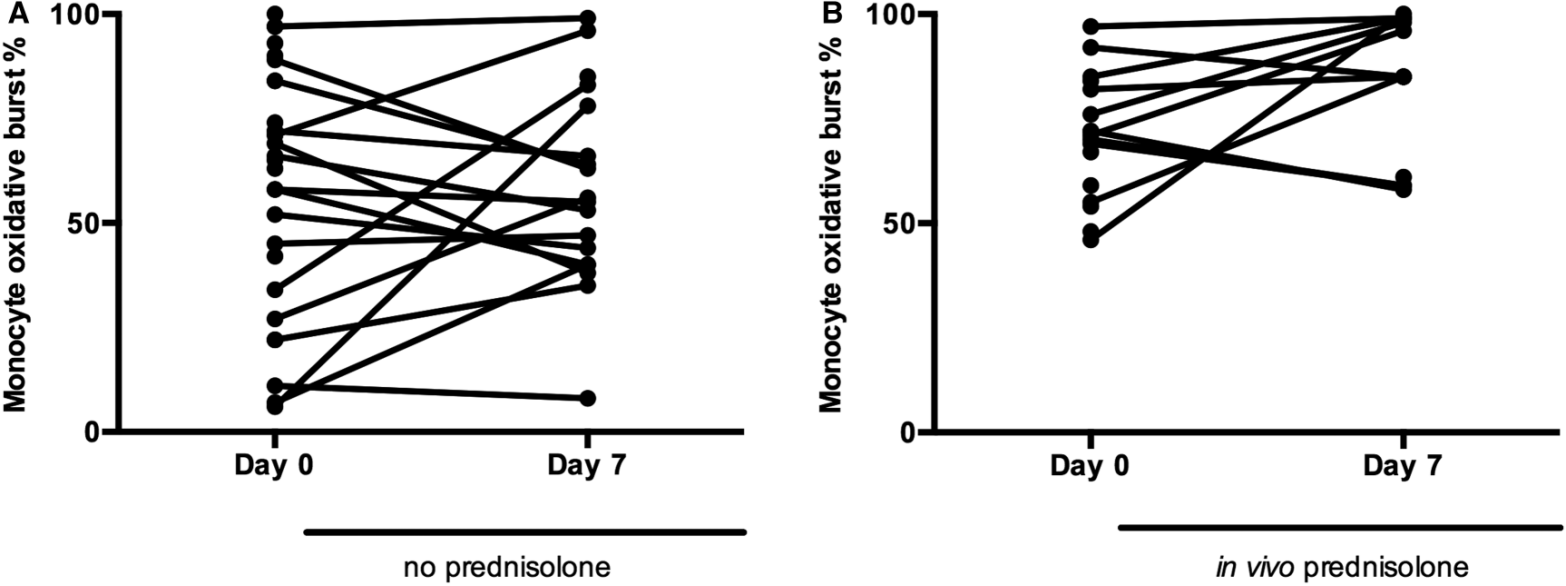
Ex vivo monocyte oxidative burst (MOB) is not affected by patient therapy with prednisolone. (A). Evolution of MOB between days 0 and 7 without prednisolone therapy; (B) evolution of MOB between days 0 and 7 with prednisolone therapy.

In vitro treatment with prednisolone did not alter MOB (*E. coli* vs *E. coli+*prednisolone: 564 vs 458 MFI; p=0.75). This was confirmed by in vivo data showing that 7 days prednisolone therapy had no effect on ex vivo phagocytosis or MOB (figure 6A, B).

### Mechanism of MOB defect

We sought to determine why monocytes from patients with SAH were unable to an adequate oxidative burst response to *E. coli*.

### Nicotinamide adenine dinucleotide phosphate substrate provision

First, we focused on the substrate of the key enzyme nicotinamide adenine dinucleotide phosphate (NADPH) oxidase. NADPH is required by NADPH oxidase to generate superoxide and effect bacterial killing. The major source of intracellular NADPH is from the pentose phosphate pathway, and more specifically the G6PDH enzyme. We tested whether G6PDH dysfunction, resulting in inadequate generation of NADPH substrate, could be the cause of defective MOB.

However, the capacity of intracellular G6PDH in generating NADPH was equivalent between patients with HC and SAH, indicating that substrate availability for the NADPH oxidase enzyme is adequate in monocytes with defective MOB (17 vs 16 nmol/min/mL; p=0.6).

### Diminished NADPH oxidase expression in monocytes with MOB defect

**Figure 7:**
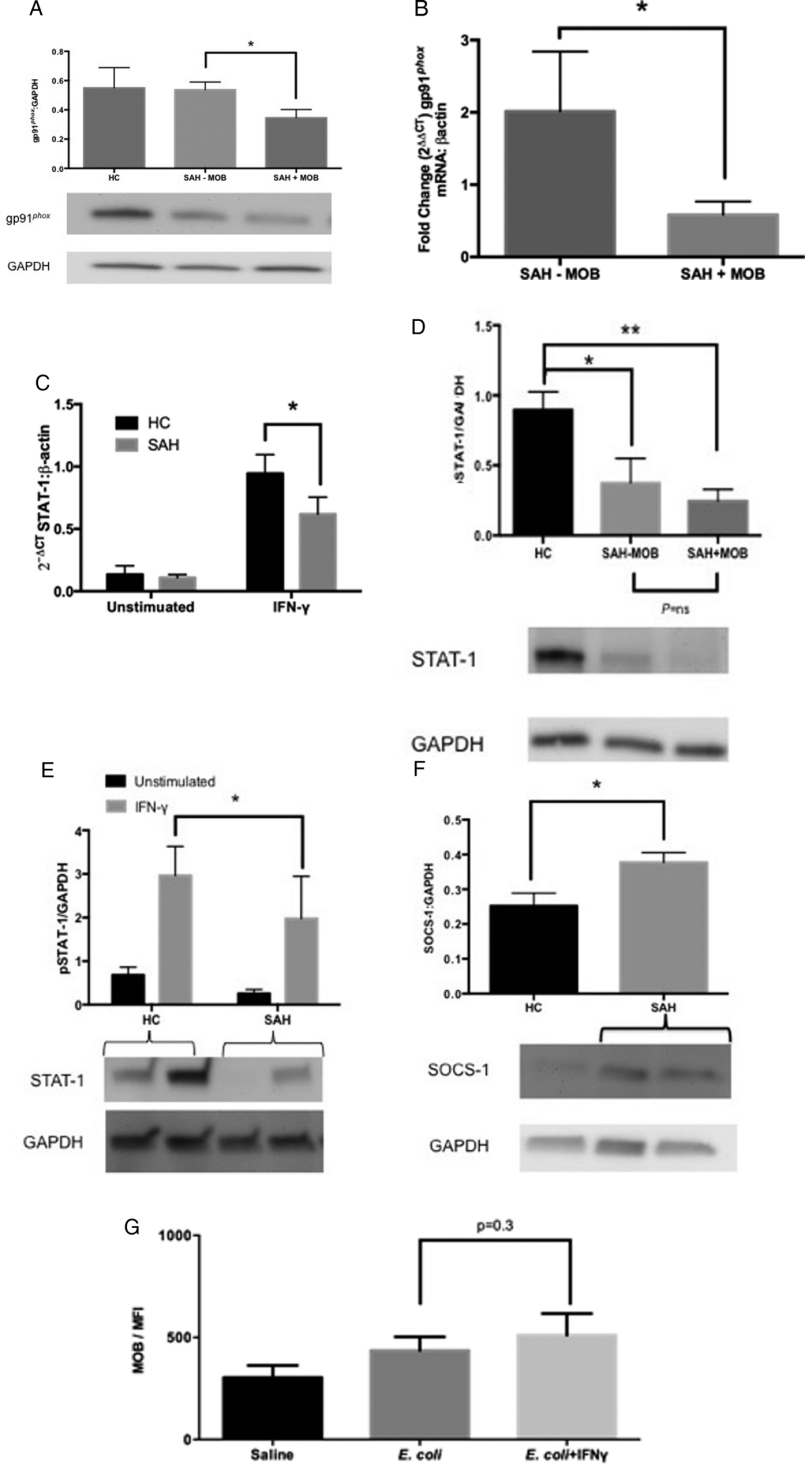
Reduced nicotinamide adenine dinucleotide phosphate (NADPH) oxidase is associated with monocyte oxidative burst (MOB) defect and aberrant Janus Kinase (JAK)-signal transducer and activator of transcription (STAT) signalling with resistance to exogenous interferon (IFN)-γ. (A) Patients with severe alcoholic hepatitis (SAH) with MOB defect (SAH+MOB) have diminished levels of the gp91^phox^ subunit of the NADPH oxidase complex compared with patients with SAH without MOB defect (SAH-MOB); (B) reduced gene expression of gp91^phox^ after IFN-γ stimulation in SAH+MOB, but not SAH-MOB, monocytes; (C) reduced gene expression of STAT-1 after IFN-γ stimulation in all SAH monocytes; (D) resting levels of phosphorylated STAT-1 are reduced in all SAH monocytes with or without MOB defect; (E) impaired activation of STAT-1 in response to IFN-γ in all SAH monocytes; (F) suppressor of cytokine signalling-1 (SOCS-1) protein is present at high levels in all SAH monocytes compared with healthy control (HC); (G) exogenous IFN-γ stimulation does not improve MOB in vitro.

gp-91*^phox^* is the major subunit of the NADPH oxidase complex and p47*^phox^* the key regulatory subunit. Intracellular monocyte expression of gp-91*^phox^* and p47*^phox^* in patients with SAH was, therefore, evaluated. Levels of p47^*phox*^ were equivalent (see online supplementary figure S3), but the level of gp91*^phox^* in the monocytes of patients with SAH with MOB defect (SAH+MOB) was significantly reduced compared with patients with SAH without MOB defect (SAH-MOB) by western blotting, (figure 7A). RT-PCR confirmed impaired gene expression of gp91*^phox^* after IFN-γ stimulation in the monocytes of patients with MOB defect compared with SAH monocytes without MOB defect (figure 7B).

### SAH monocytes are refractory to IFN-γ, which may be explained by elevated SOCS-1 protein

IFN-γ is a key cytokine involved in the stimulation of phagocyte oxidative burst. IFN-γ binding to IFN-γ receptor (IFN-γR) triggers a Janus Kinase (JAK) signalling cascade that results in phosphorylation of STAT-1,^17^ and activation of the NADPH oxidase complex. Accordingly, IFN-γ gene knockout renders mice susceptible to intracellular infections.^17^ In addition, patients suffering from CGD, in which mutations in gp91*^phox^* may be inherited, gain effective prophylaxis from opportunistic infection by treatment with subcutaneous IFN-γ.^7^ We, therefore, evaluated whether exogenous IFN-γ administration could restore defective in vitro MOB in patients with SAH.

Serum levels of IFN-γ and interleukin-12 were similar between SAH and HC (see online supplementary figure S2A, B). Expression of IFN-γR1 was higher on SAH compared with HC monocytes (1049 vs 928 MFI; p=0.05). However, gene expression of total STAT-1 in response to IFN-γ was diminished in all SAH monocytes with or without MOB (figure 7C). Similarly, protein expression of activated, phosphorylated STAT-1 with or without IFN-γ stimulation was reduced in all patients with SAH (figure 7D, E).

The JAK-STAT signalling cascade, initiated by IFN-γ binding to IFN-γR, is negatively regulated by SOCS-1.^18^ Previously, in vitro studies from healthy volunteers had shown that high levels of SOCS-1 can be induced within monocytes in response to alcohol drinking, potentially rendering monocytes refractory to IFN-γ stimulation.^18^ Indeed in the current study, western blots demonstrated increased expression of this negative intracellular regulator of STAT-1 signalling,^17^ in SAH monocytes (figure 7F). It should be noted that increased SOCS-1 and impaired activation of STAT-1 was demonstrated in monocytes from all patients with SAH, regardless of whether there was an MOB defect or not. Consistent with these findings, SAH monocytes were resistant to 24 h stimulation with IFN-γ in vitro (figure 7G).

## Discussion

In the recent STOPAH trial, 24% of deaths in SAH were attributed to infection.^16^ This highlights the importance of infection and impaired immunity for patients with SAH. The relationship between the immunodeficiency associated with SAH and susceptibility to infection is undoubtedly complex. Our study, however, reveals that SAH monocytes are characterised by normal phagocytosis with profoundly impaired oxidative burst in a subset of patients who are then demonstrably more likely to contract infection, particularly by catalase-positive organisms.

The results indicate a wide spectrum of oxidative burst capacity in patients with SAH that overlaps the range of oxidative burst capacity observed in healthy volunteers. This is not surprising, given that there are a substantial proportion of patients with SAH who do not develop infection. In this regard, we are particularly encouraged by the close correlation of defective ex vivo MOB and susceptibility to infection within the subsequent 2 weeks. The strong positive predictive value of MOB for predicting the development of infection found in the current study offers a potential biomarker to rationalise prophylactic antibiotic prescribing and reduce the incidence of infection in SAH.

Although the data linking defective MOB to susceptibility to infection are compelling, we are not able to provide direct evidence of causation. The absence of adequate animal models of alcoholic hepatitis is an obstacle to testing such hypotheses, and in vivo human studies to confirm causation require an agent that will reliably reverse defective oxidative burst. While IFN-γ is an attractive therapeutic candidate to restore MOB, data presented in the current study suggest that the efficacy that this drug has shown in the treatment of CGD,^7^ and tuberculosis,^19^ ^20^ is unlikely to extend to patients with SAH.

Resistance to interferon therapy that is mediated by SOCS-1 is also seen in hepatocytes infected with hepatitis C virus.^21^ Elsewhere, elevations in SOCS-1 have been implicated in a broad range of other persistent intracellular infections including mycobacterium tuberculosis and group A streptococcus.^22–27^ In these infections, the pathogen has evolved to hijack this important negative regulator of JAK-STAT signalling^28^ in order to subvert bacterial killing within the innate immune cell, resulting in impaired pathogen clearance.

Liver function was found not to correlate with MOB in patients with SAH. Indeed, the relationship between liver function and susceptibility to infection in SAH remains controversial.^2^ Recent clinical studies suggest that susceptibility to infection in SAH is independent of liver function,^29^ and our data of MOB are consistent with this conclusion. Bernsmeier *et al*^8^ showed that, in a group of patients with decompensated liver disease with similar liver function to patients with SAH but without alcoholic hepatitis, MOB responses to *E. coli* were preserved.

In contrast, the link between alcoholism per se and defective immunity to facultative intracellular organisms is more established.^30^ Specifically, infections by bacteria able to use catalase as a defence against phagocyte oxidative burst, known as catalase-positive organisms, are known to be prevalent in alcoholic patients,^31–33^ and this was verified in the current study. The commonest nosocomial infection in recent studies of patients with SAH is pneumonia, which is often culture negative. Nosocomial pneumonia is most often caused by Gram-negative bacilli and *Staphylococcus aureus*,^34^ both of which are catalase-positive organisms. In addition, there appears to be a preponderance of pneumonia caused by *Haemophilus influenza* and *Klebsiella pneumoniae* bacteria in alcoholic patients*,* both of which are also catalase positive*.*^35^ Of note, the association between alcoholism and the risk of contracting tuberculosis infection is frequently cited and appears to be independent of socio-economic status.^36^

The molecular cause of this increased incidence of intracellular infections in alcoholic patients remains elusive, however. Norkina *et al*^18^ found elevations in intracellular monocyte SOCS-1 and a corresponding decrease in monocyte STAT-1 activation after healthy volunteers had consumed ethanol. In line with these data, in the present study we found that alcohol drinking compensated cirrhotic patients demonstrated defective MOB but abstinent compensated cirrhotic patients did not. This suggests that heavy drinking before patients with SAH are admitted to hospital may contribute to impaired MOB.

Alcohol alone appears insufficient to disrupt MOB, however. All patients with SAH will have drunk large amounts of alcohol immediately prior to admission, and yet only a proportion present with defective MOB. Indeed in the current study, impaired activation of STAT-1 in SAH monocytes was observed whether or not the patient displayed an MOB defect ex vivo. Diminished expression of gp91*^phox^*, however, was only demonstrated in patients with defective MOB. It is likely, therefore, that impaired IFN-γ signal transduction only partially explains the observed reduction in gp91*^phox^* expression and other mechanisms, which remain to be identified, contribute to the clinical phenotype of impaired oxidative burst and increased susceptibility to infection.

Seven days of oral prednisolone had no effect on MOB. Kaufmann *et al*^37^ similarly found that in patients suffering from septic shock neutrophil phagocytosis was unaffected by 24 h intravenous hydrocortisone therapy. Our study does not, therefore, purport to explain the increased incidence of infection seen in patients treated with prednisolone in the recent STOPAH trial.

We conclude by noting that in a recent study involving >700 000 hospitalisations of cirrhotic patients in the USA, sepsis was the only cause of death that is continuing to rise.^38^ The defects of phagocytosis in SAH monocytes that have been demonstrated in the current study are, therefore, pertinent. Importantly, the mechanism that we have revealed, involving impaired transduction of IFN-γ signalling and reduced expression of NADPH oxidase, renders monocytes refractory to exogenous IFN-γ therapy. Future work should confirm MOB as a valid biomarker of susceptibility to infection in SAH and identify molecular targets that are amenable to therapeutic intervention.

## References

[1] Maddrey WC, Boitnott JK, Bedine MS, et al Corticosteroid therapy of alcoholic hepatitis. Gastroenterology 1978;75:193–9. 352788

[2] Louvet A, Wartel F, Castel H, et al Infection in patients with severe alcoholic hepatitis treated with steroids: early response to therapy is the key factor. Gastroenterology 2009;137:541–8. 10.1053/j.gastro.2009.04.062 19445945

[3] Rajkovic IA, Williams R Abnormalities of neutrophil phagocytosis, intracellular killing and metabolic activity in alcoholic cirrhosis and hepatitis. Hepatology 1986;6:252–62. 10.1002/hep.1840060217 3007318

[4] Mookerjee RP, Stadlbauer V, Lidder S, et al Neutrophil dysfunction in alcoholic hepatitis superimposed on cirrhosis is reversible and predicts the outcome. Hepatology 2007;46:831–40. 10.1002/hep.21737 17680644

[5] Rolas L, Makhezer N, Hadjoudj S, et al Inhibition of mammalian target of rapamycin aggravates the respiratory burst defect of neutrophils from decompensated patients with cirrhosis. Hepatology 2013;57:1163–71. 10.1002/hep.26109 23080369

[6] Markwick LJL, Riva A, Ryan JM, et al Blockade of PD1 and TIM3 restores innate and adaptive immunity in patients with acute alcoholic hepatitis. Gastroenterology 2015;148:590–602.e10. 10.1053/j.gastro.2014.11.041 25479137

[7] Ezekowitz, et al A controlled trial of interferon gamma to prevent infection in chronic granulomatous disease. N Engl J Med 1991;324:509–16. 1846940 10.1056/NEJM199102213240801

[8] Bernsmeier C, Pop OT, Singanayagam A, et al Patients with acute-on-chronic liver failure have increased numbers of regulatory immune cells expressing the receptor tyrosine kinase MERTK. Gastroenterology 2015;148:603–15.e14. 10.1053/j.gastro.2014.11.045 25479139

[9] Antoniades CG, Berry PA, Davies ET, et al Reduced monocyte HLA-DR expression: a novel biomarker of disease severity and outcome in acetaminophen-induced acute liver failure. Hepatology 2006;44:34–43. 10.1002/hep.21240 16799971

[10] Antoniades CG, Quaglia A, Taams LS, et al Source and characterization of hepatic macrophages in acetaminophen-induced acute liver failure in humans. Hepatology 2012;56:735–46. 10.1002/hep.25657 22334567

[11] Forrest E, Mellor J, Stanton L, et al Steroids or pentoxifylline for alcoholic hepatitis (STOPAH): study protocol for a randomised controlled trial. Trials 2013;14:262 10.1186/1745-6215-14-262 23958271 PMC3766225

[12] Bajaj JS, O'Leary JG, Reddy KR, et al Second infections independently increase mortality in hospitalized patients with cirrhosis: the north American Consortium for the study of end-stage liver disease (NACSELD) experience. Hepatology 2012;56:2328–35. 10.1002/hep.25947 22806618 PMC3492528

[13] O'Brien AJ, Fullerton JN, Massey KA, et al Immunosuppression in acutely decompensated cirrhosis is mediated by prostaglandin E2. Nat Med 2014;20:518–23. 10.1038/nm.3516 24728410 PMC5369639

[14] Decleva E, Menegazzi R, Busetto S, et al Common methodology is inadequate for studies on the microbicidal activity of neutrophils. J Leukoc Biol 2006;79:87–94. 10.1189/jlb.0605338 16244110

[15] Louvet A, Naveau S, Abdelnour M, et al The Lille model: a new tool for therapeutic strategy in patients with severe alcoholic hepatitis treated with steroids. Hepatology 2007;45:1348–54. 10.1002/hep.21607 17518367

[16] Thursz MR, Richardson P, Allison M, et al Prednisolone or pentoxifylline for alcoholic hepatitis. N Engl J Med 2015;372:1619–28. 10.1056/NEJMoa1412278 25901427

[17] Schroder K, Hertzog PJ, Ravasi T, et al Interferon-gamma: an overview of signals, mechanisms and functions. J Leukoc Biol 2004;75:163–89. 10.1189/jlb.0603252 14525967

[18] Norkina O, Dolganiuc A, Catalano D, et al Acute alcohol intake induces SOCS1 and SOCS3 and inhibits cytokine-induced STAT1 and STAT3 signaling in human monocytes. Alcohol Clin Exp Res 2008;32:1565–73. 10.1111/j.1530-0277.2008.00726.x 18616672 PMC4116614

[19] Condos R, Rom WN, Schluger NW Treatment of multidrug-resistant pulmonary tuberculosis with interferon-gamma via aerosol. Lancet 1997;349:1513–15. doi:S0140-6736(96)12273-X 9167461 10.1016/S0140-6736(96)12273-X

[20] Dawson R, Condos R, Tse D, et al Immunomodulation with recombinant interferon-gamma1b in pulmonary tuberculosis. PLoS ONE 2009;4:e6984 10.1371/journal.pone.0006984 19753300 PMC2737621

[21] Read SA, Tay ES, Shahidi M, et al The mechanism of interferon refractoriness during hepatitis C virus infection and its reversal with a peroxisome proliferator-activated receptor α agonist. J Interferon Cytokine Res 2015;35:488–97. 10.1089/jir.2014.0209 25734487 PMC4490708

[22] Carow B, Ye XQ, Gavier-Wide D, et al Silencing suppressor of cytokine signaling-1 (SOCS1) in macrophages improves Mycobacterium tuberculosis control in an interferon-γ (IFN-γ)-dependent manner. J Biol Chem 2011;286:26873–87. 10.1074/jbc.M111.238287 21622562 PMC3143647

[23] Srivastav S, Ball WB, Gupta P, et al Leishmania donovani prevents oxidative burst-mediated apoptosis of host macrophages through selective induction of suppressors of cytokine signaling (SOCS) proteins. J Biol Chem 2014;289:1092–105. 10.1074/jbc.M113.496323 24275663 PMC3887177

[24] Zimmermann S, Murray PJ, Heeg K, et al Induction of suppressor of cytokine signaling-1 by Toxoplasma gondii contributes to immune evasion in macrophages by blocking IFN-gamma signaling. J Immunol 2006;176:1840–7. 10.4049/jimmunol.176.3.1840 16424215

[25] Wu J, Ma C, Wang H, et al A MyD88–JAK1–STAT1 complex directly induces SOCS-1 expression in macrophages infected with Group A Streptococcus. Cell Mol Immunol 2015;12:373–83. 10.1038/cmi.2014.107 25399770 PMC4654310

[26] Aslam B, Ahmad J, Ali A, et al On the modelling and analysis of the regulatory network of dengue virus pathogenesis and clearance. Comput Biol Chem 2014;53:277–91. 10.1016/j.compbiolchem.2014.10.003 25462335

[27] Kundu K, Dutta K, Nazmi A, et al Japanese encephalitis virus infection modulates the expression of suppressors of cytokine signaling (SOCS) in macrophages: Implications for the hosts’ innate immune response. Cell Immunol 2013;285:100–10. 10.1016/j.cellimm.2013.09.005 24140964

[28] Greenhalgh CJ, Miller ME, Hilton DJ, et al Suppressors of cytokine signaling: Relevance to gastrointestinal function and disease. Gastroenterology 2002;123:2064–81. 10.1053/gast.2002.37068 12454862

[29] Rudler M, Mouri S, Charlotte F, et al Prognosis of treated severe alcoholic hepatitis in patients with gastrointestinal bleeding. J Hepatol 2015;62:816–21. 10.1016/j.jhep.2014.11.003 25450199

[30] Jerrells TR, Sibley D Effects of ethanol on cellular immunity to facultative intracellular bacteria. Alcohol Clin Exp Res 1995;19:11–16. 10.1111/j.1530-0277.1995.tb01466.x 7539599

[31] Storch G, Baine WB, Fraser DW, et al Sporadic community-acquired Legionnaires’ disease in the United States. A case-control study. Ann Intern Med 1979;90:596–600. 434642 10.7326/0003-4819-90-4-596

[32] Forsblom E, Ruotsalainen E, Mölkänen T, et al Predisposing factors, disease progression and outcome in 430 prospectively followed patients of healthcare- and community-associated Staphylococcus aureus bacteraemia. J Hosp Infect 2011;78:102–7. 10.1016/j.jhin.2011.03.010 21511366

[33] Guevara RE, Mascola L, Sorvillo F Risk factors for mortality among patients with nonperinatal listeriosis in Los Angeles County, 1992–2004. Clin Infect Dis 2009;48:1507–15. 10.1086/598935 19400687

[34] Ellison RT, Donowitz GR. Acute Pneumonia. In: Bennett J, Dolin R, Blaser M, eds. Mandell, Douglas and Bennett's Principles and practice of infectious diseases: 8th edition, pp 823–47.

[35] Jong GM, Hsiue TR, Chen CR, et al Rapidly fatal outcome of bacteremic Klebsiella pneumoniae pneumonia in alcoholics. Chest 1995;107:214–17. 10.1378/chest.107.1.214 7813281

[36] Lonnroth K, Williams BG, Stadlin S, et al Alcohol use as 289., arisk factor for tuberculosis; a systematic review. BMC Public Health 2008;8 10.1186/1471-2458-8-289 PMC2533327 18702821

[37] Kaufmann I, Briegel J, Schliephake F, et al Stress doses of hydrocortisone in septic shock: Beneficial effects on opsonization-dependent neutrophil functions. Intensive Care Med 2008;34:344–9. 10.1007/s00134-007-0868-8 17906853

[38] Schmidt ML, Barritt a. S, Orman ES, et al Decreasing Mortality Among Patients Hospitalized With Cirrhosis in the United States From 2002 Through 2010. Gastroenterology 2015;148:967–77.e2. 10.1053/j.gastro.2015.01.032 25623044 PMC4430328

