# Supplementary material for: Defective monocyte oxidative burst predicts infection in alcoholic hepatitis and is associated with reduced expression of NADPH oxidase
Source: Gut. 2016 Feb 9;66(3):519–29. doi: 10.1136/gutjnl-2015-310378 (PMC5534772; doi:10.1136/gutjnl-2015-310378)

Supplementary tables:

Supplementary table 1: criteria for study entry

| | |
|---|---|
| **Inclusion Criteria** | • >18 years old<br>• Serum bilirubin 80mmol/L<br>• Alcohol consumption >80g/day (men) >60g/day (women)<br>• <4 weeks since admission to hospital<br>• Maddrey's discriminant function ≥ 32 |
| **Exclusion criteria** | • > 2 months abstinence<br>• > 3 months prior jaundice<br>• No evidence Hepatitis B nor C infection, hepatocellular carcinoma, nor biliary obstruction<br>• Pregnant or lactating women<br>• Serum creatinine >500mmol/L<br>• Current malignancy<br>• AST > 500 or ALT >300<br>• Untreated sepsis |

Supplementary table 2: infections acquired within two weeks of sampling

| Infection | Number of cases |
|---|---|
| Pneumonia | 10 |
| Urinary tract infection | 3 |
| Cellulitis | 1 |
| Tuberculosis | 1 |
| Clostridium difficile | 1 |
| Necrotising Fasciitis | 1 |

Supplementary figures:

Supplementary figure 1: A. staining with live/dead marker 7-AAD shows few dead cells; B. FACS dot plot demonstrating monocyte purity after monocyte isolation by negative selection

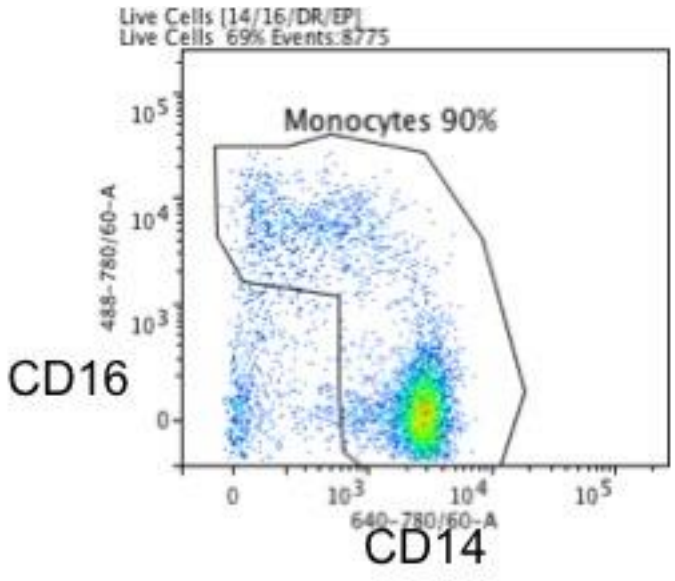


Supplementary figure 2: A. serum IFN-?; B. serum IL-12 in SAH vs HC and CLD.1

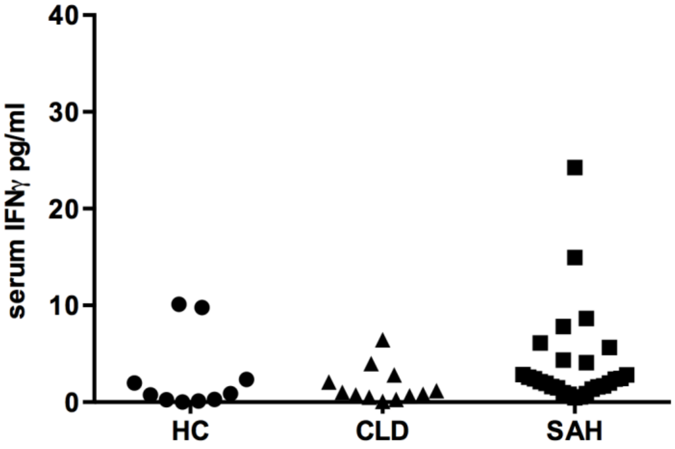


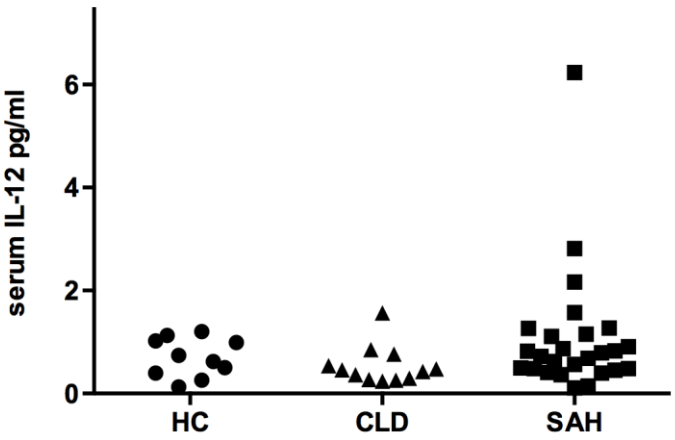

Supplementary figure 3: expression of p47phox protein in SAH monocytes with an without MOB defect by Western blotting

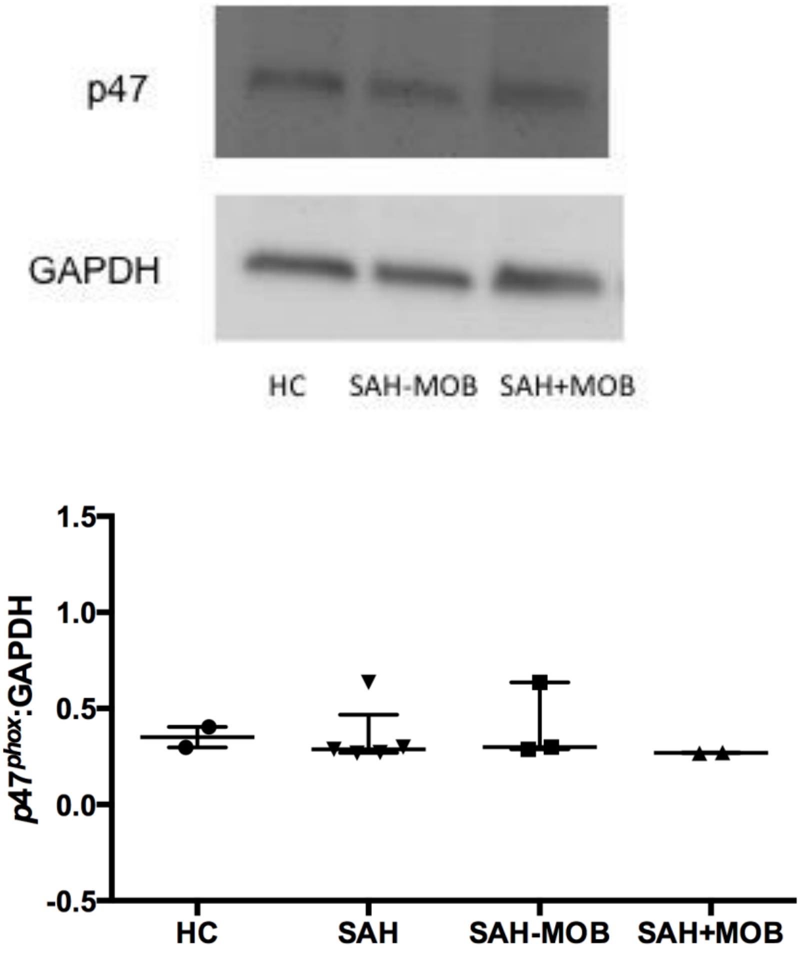

Supplement: Supplementary materials [file gutjnl-2015-310378supp001.pdf]
