# Supplementary material for: Defective monocyte oxidative burst predicts infection in alcoholic hepatitis and is associated with reduced expression of NADPH oxidase
Source: Gut. 2016 Feb 9;66(3):519–29. doi: 10.1136/gutjnl-2015-310378 (PMC5534772; doi:10.1136/gutjnl-2015-310378)
Supplement: Supplementary materials [file gutjnl-2015-310378supp002.pdf]

Clinical, haematological and biochemical parameters

Full blood count, international normalised ratio (INR) and prothrombin time, liver and renal function tests, C-reactive protein (CRP) and clinical variables including the prescription of intravenous antibiotics were prospectively entered into a database. Maddrey's discriminant function (MDF), model for end-stage liver disease (MELD), Lille score and early change in bilirubin level (ECBL) were calculated.

Monocyte phenotyping

Fluorochrome conjugated monoclonal antibodies against CD14, CD163, CD64, CD206, CD34 and IFNγR1 (eBioscience, UK) were used to determine expression of phenotypic markers on monocytes from whole blood using flow-cytometry as previously described,[8]. Results are expressed as mean fluorescence intensity (MFI). Flow cytometry data was analysed using Flowlogic software (Inivai Technologies Pty Ltd, Australia).

Serum cytokines

Serum IFN-γ and IL-12 concentrations were measured by the Mesoscale Discovery detection multiplex system (Gaithersburg, US).

Phagocytosis assay

Phagocytosis by $CD14^+$ monocytes in whole blood was assessed *ex vivo*. The ability of monocyte subsets to phagocytose *E. coli* was measured using a modified flow cytometry-based pHrodo® assay according to the manufacturers instructions (Life Technologies, UK).

Measurement of intracellular G6PDH function

Isolated monocytes were lysed using NP-40 buffer and intracellular enzyme function was immediately measured using the Glucose-6 Phosphate Dehydrogenase (G6PDH) Assay Kit according to the manufacturer's instructions (Abcam, UK).

Real-time polymerase chain reaction measurement of gp-91^{phox} and STAT-1 gene expression

Total RNA from isolated monocytes was extracted using the RNeasy Mini Kit (Qiagen, Germany). Complementary DNA was synthesised from RNA using the RETROscript Kit (Life Technologies, US). Quantitative real-time PCR (RT-PCR) was performed using TaqMan Gene Expression assays specific for STAT-1 (Hs01013996_m1), SOCS-1 (Hs00864158_g1) and gp-91^{phox} (Hs00166163_m1) (Life Technologies, US). Beta actin was used as the endogenous control (Hs99999903_m1) (Life Technologies, US). Assays were performed on a StepOne Real-Time PCR System (Applied Biosciences, UK).
